# Prognosis of ovarian cancer in women with type 2 diabetes using metformin and other forms of antidiabetic medication or statins: a retrospective cohort study

**DOI:** 10.1186/s12885-018-4676-z

**Published:** 2018-07-28

**Authors:** Elina Urpilainen, Mikko Marttila, Ari Hautakoski, Martti Arffman, Reijo Sund, Pirjo Ilanne-Parikka, Reetta Arima, Jenni Kangaskokko, Ulla Puistola, Marianne Hinkula, Esa Läärä

**Affiliations:** 1 Department of Obstetrics and Gynecology, PEDEGO Research Unit, Medical Research Center Oulu, University of Oulu and University Hospital of Oulu, P.O. Box 23, FIN-90029 Oulu, Finland; 2 Children, Adolescents and Families Unit, Department of Welfare, National Institute for Health and Welfare, P.O. Box 310, FIN-90101 Oulu, Finland; 3 Research Unit of Mathematical Sciences, University of Oulu, P.O. Box 8000, FIN-90014 Oulu, Finland; 4 Service System Research Unit, National Institute for Health and Welfare, P.O. Box 30, FIN-00271 Helsinki, Finland; 5 Centre for Research Methods, Department of Social Research, University of Helsinki, Helsinki, Finland; 6 Institute of Clinical Medicine, University of Eastern Finland, P.O. Box 1627, FIN-70211 Kuopio, Finland; 7 The Diabetes Center, Finnish Diabetes Association, FIN-33680 Tampere, Finland; 8 Medical Research Center Oulu, University of Oulu, P.O. Box 8000, FIN-90014 Oulu, Finland; 9 Coronaria Diagnostics Oy, Oulu laboratory, FIN-90100 Oulu, Finland

**Keywords:** Ovarian cancer, Cancer survival, Cancer prognosis, Type 2 diabetes, Metformin, Statins, Antidiabetic medication

## Abstract

**Background:**

Ovarian cancer is one of the most lethal cancers and women with type 2 diabetes (T2D) have even poorer survival from it. We assessed the prognosis of ovarian cancer in women with type 2 diabetes treated with metformin, other forms of antidiabetic medication, or statins.

**Methods:**

Study cohort consisted of women with T2D diagnosed with ovarian cancer in Finland 1998–2011. They were identified from a nationwide diabetes database (FinDM), being linked to several national registers. Patients were grouped according to their medication in the three years preceding ovarian cancer diagnosis. The Aalen–Johansen estimator was used to describe cumulative mortality from ovarian cancer and from other causes in different medication groups. Mortality rates were analysed by Cox models, and adjusted hazard ratios (HR) with 95% confidence intervals (95% CIs) were estimated in relation to the use of different forms of medication. Main outcome measures were death from ovarian cancer and death from other causes.

**Results:**

During the accrual period 421 newly diagnosed ovarian cancers were identified in the FinDM database. No evidence was found for any differences in mortality from ovarian cancer or other causes between different antidiabetic medication groups. Pre-diagnostic use of statins was observed to be associated with decreased mortality from ovarian cancer compared with no such use (HR 0.72, 95% CI 0.56–0.93).

**Conclusions:**

Our findings are inconclusive as regards the association between metformin and ovarian cancer survival. However, some evidence was found for improved prognosis of ovarian cancer with pre-diagnostic statin use, requiring cautious interpretation, though.

**Electronic supplementary material:**

The online version of this article (10.1186/s12885-018-4676-z) contains supplementary material, which is available to authorized users.

## Background

Ovarian cancer (OC) is one of the most lethal cancers, causing 140,000 deaths annually worldwide [1]. The high mortality rate is attributed to the fact that women present with the disease at a late stage, as the symptoms are unspecific and do not emerge until the cancer is advanced [2]. Standard treatment includes cytoreductive surgery and adjuvant chemotherapy with platinum and taxane-based cytostatics. In early disease, treatment with chemotherapy can be curative but in advanced ovarian cancer, most patients will have a recurrent disease within 18 months [3].

Women with type 2 diabetes (T2D) are reported to have poorer survival from OC compared with those without T2D [4]. Metformin is a type of oral antidiabetic medication recommended as first-line treatment in T2D [5]. In some previous studies its use has been linked to favourable survival in cases of OC [6–8]. Other studies have not been able to find an association between metformin use and better prognosis of OC in women with T2D [4]. The main problem in previous studies is the small number of patients.

Metformin has anti-mitotic, anti-angiogenic and anti-inflammatory properties [9]. It inhibits growth of OC cells in a time- and dose-dependent way, and inhibition is also seen in platinum-resistant cell lines [10]. Preclinical in vivo studies have suggested that metformin-treated mice develop smaller ovarian tumours and fewer metastatic nodules than controls [11]. It has also been shown that metformin decreases proliferation of OC cells, decreases angiogenesis and potentiates the cytotoxic effect of cisplatin [12].

Patients with T2D have an elevated risk of cardiovascular diseases and hypercholesterolaemia, and are widely treated with statins. In Finland, 40% of patients diagnosed with T2D have been found to use lipid-lowering medication without diagnosis of coincident coronary heart disease, and the percentage of medication users increases to 73% in patients with T2D having coronary heart disease [13]. Statins (HMG-CoA [3-hydroxy-3-methylglutaryl-CoA] reductase inhibitors) block formation of cholesterol by inhibiting HMG-COA conversion to mevalonate [14]. Both in vitro and in vivo studies indicate that statins have antiproliferative, proapoptotic, anti-invasive and radio-sensitizing effects [15].

In most previous reports, OC patients who used statins showed better overall survival [16–18]. However, in a large population-based study by Nielsen et al., statin use predicted reduced cancer-specific mortality among all cancer patients. No sufficient evidence for improved prognosis was found when investigating OC patients alone [19]. Also, Habis et al. found no difference between statin users and non-users as regards OC survival [20].

In the present nationwide register-based cohort study the associations between use of metformin, other types of antidiabetic medication and statins, and the prognosis of OC in patients with T2D was evaluated.

## Methods

### Study population and design

STROBE guidelines for observational studies were followed in writing this report [21]. The data on people with diabetes were collected from a Finnish diabetes database (FinDM), which combines information from several nationwide registers including the Care Register for Health Care and the Finnish Hospital Discharge Register of the National Institute for Health and Welfare, the Causes of Death Statistics of Statistics Finland and the Register on Medical Special Reimbursements and the Register on Reimbursed Drug purchases of the Social Insurance Institution [22].

The FinDM database includes about 244,000 women with prevalent (at the beginning of 1996, *n* = 172,000) or incident (from 1 January 1996 to 31 December 2011, *n* = 72,000) T2D. Persons with diabetes were entered in the FinDM database if they met at least one of these criteria: diagnosis of diabetes in some of the used registers (Finnish Health Care Register, the Hospital Benchmarking database, the Medical Birth Register, the Diabetes in Finland study or the Register of Causes of Death) or reimbursement for antidiabetic medication (ADM) in the register on Reimbursed Drug purchases of the Social Insurance Institution [22]. The diagnosis of type 2 diabetes is based on World Health Organization (WHO) criteria in Finland [23]. Data on diagnoses in hospital records have been available since 1969 for inpatients and since 1998 for outpatients [22]. Classification to type 1 (primarily insulin-dependent) and type 2 diabetes is based on the ADM which was used as the first-line treatment [22]. Good coverage of persons with diabetes was shown in FinDM when compared with a local diabetes register covering the Helsinki region [24]. The FinDM database holds information about the quantity and the date of purchase of all medication prescribed by doctors and reimbursed by the Social Insurance Institution, including antidiabetic and statin medication, starting from 1994 [22].

**Fig. 1:**
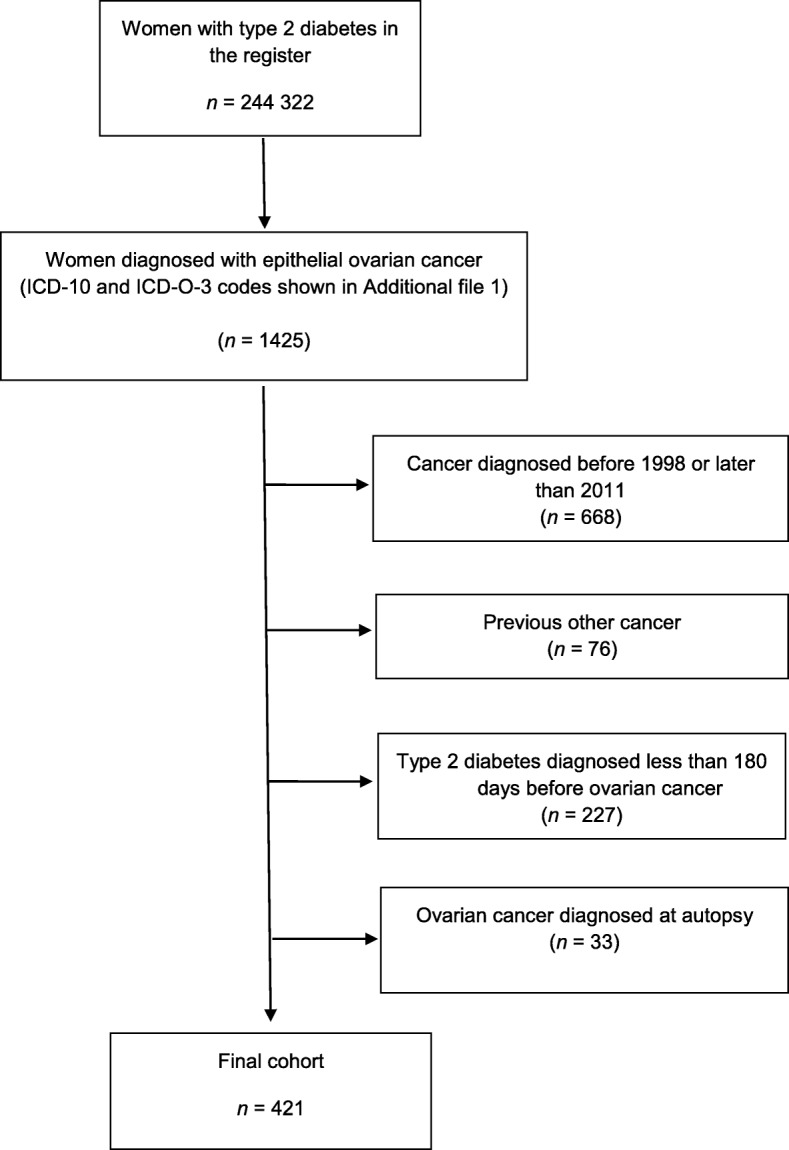
Flowchart

From the FinDM database we identified 757 women who were diagnosed with epithelial ovarian cancer between 1 January 1998 and 31 December 2011 (Fig. 1). We excluded those women with a prior cancer diagnosis (other than non-melanoma skin cancer). We included women in whom the estimated duration of T2D was at least 180 days before OC diagnosis. We further excluded those women whose ovarian cancer were diagnosed at autopsy. Data on the cancer cases, their histology and stage were obtained from the Finnish Cancer Registry (ICD-O-3 [International Classification of Diseases for Oncology, Third Edition] codes are shown in Additional file 1) [25]. Stage was categorized as local, advanced (including growth to adjacent tissues, metastasis in regional lymph nodes and distant metastasis) or unknown. The final study cohort contained 421 women with T2D, who were diagnosed with epithelial ovarian cancer at least 180 days after the diagnosis of T2D in 1998–2011 (Fig. 1).

### Exposure and covariates assessment

Patients were classified into mutually exclusive groups according to ADM purchased during the three years before OC diagnosis: metformin only, other oral ADM only, metformin and other oral ADM, insulin at any time and no history of ADM. Regardless of patients ADM use, they were also classified as statin users and non-users. The Anatomical Therapeutic Chemical (ATC) Classification System was used to define used medication. ATC codes for different types of oral ADM and statins are shown in Additional file 2. For all types of medication, exposure was considered to begin 180 days after the date of purchase. A patient was classified as a user of ADM when she had purchased metformin or other oral ADM for 180 days or longer in the three years preceding OC diagnosis, with no history of insulin purchases. If a patient had purchased oral ADM for less than 180 days, she was classified in the group “no history of antidiabetic medication”. One purchase of insulin was enough to place the patients in the “insulin ever” group. Respectively, a patient was classified as a statin user if she had purchased statin for 180 days or longer in the three years preceding OC diagnosis. The cumulative use of metformin and statins, respectively, was estimated by way of defined daily doses (DDDs) purchased within three years before diagnosis of ovarian cancer.

### Outcome ascertainment

Follow-up of the study cohort began at the date of diagnosis of ovarian cancer and ended at the time of death, emigration or closure of the follow-up on 31 December 2011, whichever happened first. Follow-up information was collected from the Finnish Cancer Registry. By using personal identity codes, the records of the Finnish Cancer Registry are annually matched with those in the Cause of Death Statistics database, which is maintained by Statistics Finland. This way, dates and causes of death (using ICD-10 [International Statistical Classification of Diseases and Related Health Problems, 10th Revision] codes) are attached to the records in the Registry. Personnel at the Finnish Cancer Registry compare the official causes of death of each patient with diagnosed cancer with all available data for that cancer, and make a judgement as to whether the patient died of that cancer or of something else. The classification of deaths into the two categories in this study, i.e. deaths from OC and deaths resulting from other causes, was based on that judgement. Data in the Finnish Cancer Registry is also linked regularly to the Central Population Register of Finland to check the correctness of personal identity codes, complete name, vital status, possible date of death or emigration and the official place of residence before the date of diagnosis [26].

### Statistical analysis

Mortality from OC and from other causes, respectively, was assessed in different medication groups by using the Aalen–Johansen estimator of the cumulative incidence function for competing risks [27, 28]. Cox proportional hazards models were fitted for the two causes of death separately to adjust for the effects of calendar year, age, duration of T2D, and stage at diagnosis of OC. Hazard ratios (HRs), with accompanying 95% confidence intervals (CIs) of related to the two causes of death between medication groups were estimated from the adjusted Cox models. In supplementary analysis, the medication group membership indicators in the Cox models were replaced with cubic spline terms for the total amount of DDDs of each type of medication purchased [29]. This allowed estimation of a potentially nonlinear dose-dependent effect of the medications on the mortality from OC. Plots of scaled Schoenfeld residuals were visually inspected [30], but no evidence for a violation of the proportional hazards assumption could be observed which would have any impact on inference. R environment version 3.3.2 was used throughout for data preparation and statistical analysis; the Cox models were fitted and assumptions checked with functions provided in the “survival” package [31, 32].

## Results

The age range in the final study cohort (*n* = 421) was 42 to 92 years at the time of OC diagnosis (Table 1). The greatest percentage (38%) of ovarian cancers were diagnosed at the ages of 70 to 79 years. The majority (78%) of OC cases were at an advanced stage at the time of diagnosis. The median duration of follow-up for a patient was 2.2 years, with a total of 1378 person-years observed in the study.

**Table 1:** Distribution of prognostic factors in different medication groups^a^

	Antidiabetic medication					Use of statins		
	Metformin^b^	Other oral ADM^b^	Metformin and other oral ADM^b^	Insulin	No use of ADM	Yes^b^	No	Total
Number of patients	77	58	100	82	104	186	235	421
Age at diagnosis, years								
Median	69	75	70	71	72	71	71	71
IQR^c^	63─77	66─80	61─77	65─78	64─79	65─77	62─78	64─78
Age categories, years (%)								
42─59	8 (10)	6 (10)	19 (19)	9 (11)	17 (16)	18 (10)	41 (17)	59 (14)
60─69	33 (43)	13 (22)	31 (31)	28 (34)	27 (26)	66 (35)	66 (28)	132 (31)
70─79	30 (39)	24 (41)	42 (42)	29 (35)	35 (34)	74 (40)	86 (37)	160 (38)
80─92	6 (8)	15 (26)	8 (8)	16 (20)	25 (24)	28 (15)	42 (18)	70 (17)
Duration of T2D, years (%)								
Median	3.1	5.0	6.2	10.8	7.0	6.3	5.7	6.2
IQR^c^	2.0─5.5	3.1─8.3	4.1─8.9	6.8─15.0	2.0─10.1	3.1─10.0	3.1─10.0	3.1─10.1
0.5 ─ < 3	37 (48)	15 (26)	13 (13)	4 (5)	34 (33)	45 (24)	58 (25)	103 (24)
3 ─ < 6	24 (31)	20 (34)	30 (30)	13 (16)	13 (12)	40 (22)	60 (26)	100 (24)
6 ─ < 12	14 (18)	19 (33)	44 (44)	30 (37)	41 (39)	71 (38)	77 (33)	148 (35)
12 ─ < 34	2 (3)	4 (7)	13 (13)	35 (43)	16 (15)	30 (16)	40 (17)	70 (17)
Stage (%)								
Local	14 (18)	6 (10)	11 (11)	11 (13)	10 (10)	24 (13)	28 (12)	52 (12)
Advanced	58 (75)	45 (78)	77 (77)	64 (78)	86 (83)	142 (76)	188 (80)	330 (78)
Unknown	5 (6)	7 (12)	12 (12)	7 (9)	8 (8)	20 (11)	19 (8)	39 (9)

^a^The entries are number and percentages (in parenthesis) if not otherwise stated

^b^Duration of medication ≥180 days

^c^Interquartile range

Eighteen per cent of the OC patients used metformin as the only antidiabetic drug, 14% used other types of oral ADM, 24% used metformin combined with other types of ADM and 19% used insulin (Table 1). A quarter of the OC patients did not have a history of ADM use. On average, metformin-only users were younger (median 69 years old) and patients who used other types of oral ADM only were older (median 75 years old) when compared with patients in other ADM groups (Table 1). The duration of diabetes was shorter in the metformin-only group (median 3.1 years) and longer in the insulin group (median 10.8 years) (Table 1). The stage distribution of ovarian cancer was similar across the ADM groups (Table 1).

One hundred and eighty-six (44%) of the OC patients were statin users. Statin users and non-users were similar as regards age distribution, duration of diabetes and OC stage (Table 1). The most commonly used statins were lipophilic statins, i.e. simvastatin (56.5% of statin users) and atorvastatin (26.9%).

**Fig. 2:**
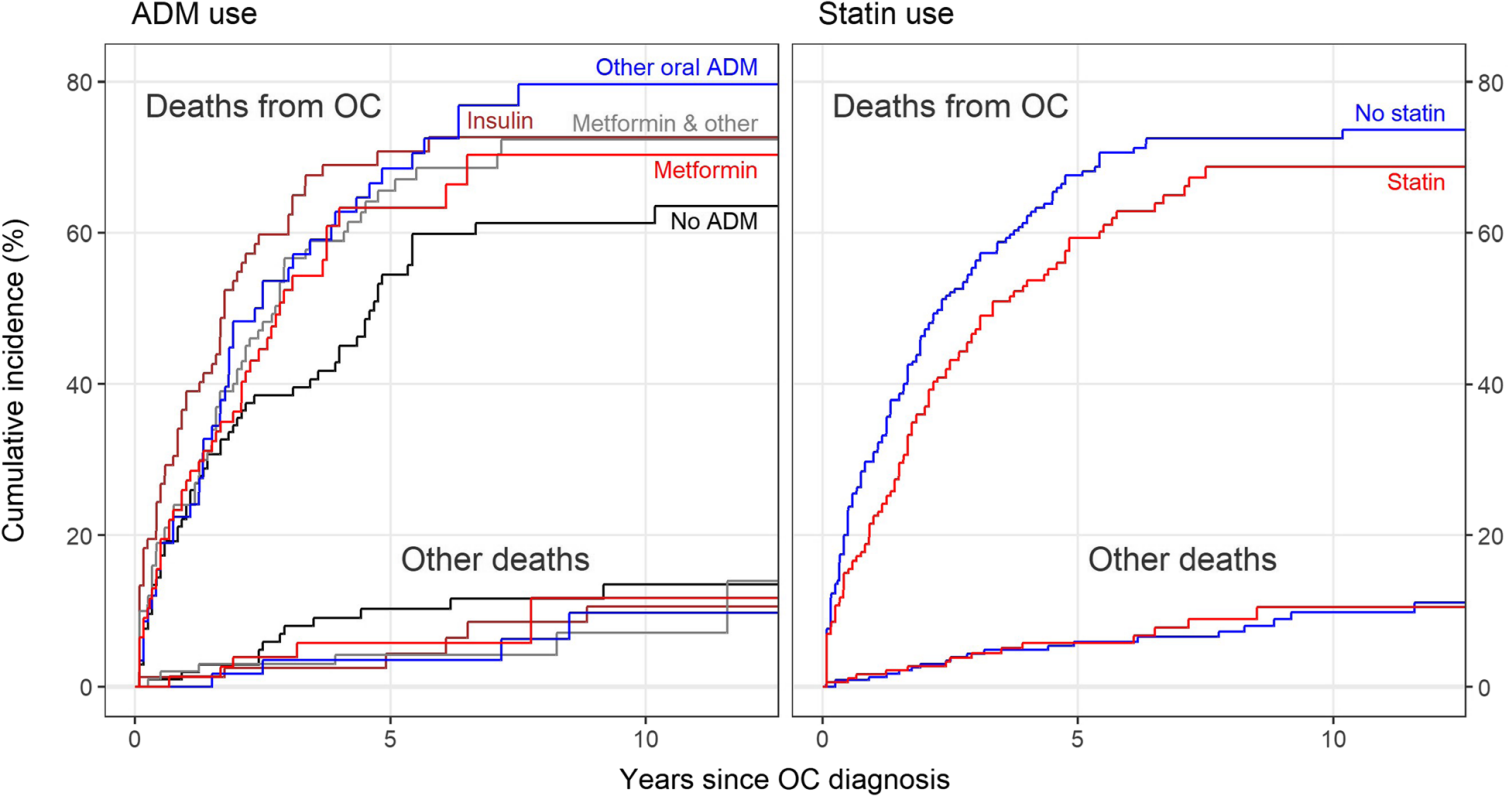
Cumulative incidence function curves of death from OC and from other causes in different groups

Three hundred and ten patients (74%) died during the follow-up period, most of them (276 patients, 89%) from ovarian cancer. Unadjusted cumulative mortality from OC by 10 years after diagnosis varied from 61 to 80% across the ADM groups and from 69 to 73% between the groups defined by statin use, whereas the mortality from other causes by 10 years was on average around 10% with less variability across various ADM and statin groups (Fig. 2). When adjusted for age, calendar year and duration of diabetes at diagnosis of OC and for stage and use of statins, the mortality from OC and from other causes were not found to differ by ADM (Table 2). Pre-diagnostic use of metformin as the only treatment for T2D had an adjusted HR of 1.15 (95% CI 0.74–1.79) for ovarian cancer death and an adjusted HR of 1.85 (95% CI 0.44–7.73) for death from other causes (Table 2), compared with use of other forms of oral ADM. Duration of diabetes was not found to be associated with mortality from ovarian cancer, nor from other causes (Table 2). Pre-diagnostic use of statin was observed to predict decreased mortality from ovarian cancer compared with no use of statin (adjusted HR 0.72, 95% CI 0.56–0.93) (Table 2). No sufficient evidence was found for cumulative use of metformin or statins (DDDs) to be associated with mortality from OC (Additional file 3). The results of Cox modelling for the association of all-cause mortality with ADM and with statins were essentially the same as those for deaths from OC (data not shown).

**Table 2:** Estimation results from Cox proportional hazard models of mortality from OC and from other causes

Variable	Mortality from OC				Mortality from other causes		
	Group size	Deaths	HR	(95% CI)	Deaths	HR	(95% CI)
Year of diagnosis							
1998─2002	115	84	1.00	Ref.	13	1.00	Ref.
2003─2007	149	106	1.17	(0.86–1.59)	12	1.16	(0.45–2.99)
2008─2011	157	86	0.97	(0.69–1.37)	9	1.13	(0.39–3.27)
Age at diagnosis (years)							
42─59	59	30	0.67	(0.44–1.04)	1	0.18	(0.02–1.53)
60─69	132	76	1.00	Ref.	9	1.00	Ref.
70─79	160	120	1.53	(1.14–2.05)	14	2.49	(1.03–6.05)
80─92	70	50	2.88	(1.98–4.20)	10	5.40	(1.99–14.65)
Duration of diabetes (years)							
0.5─ < 3	103	61	1.00	Ref.	11	1.00	Ref.
3─ < 6	100	70	1.31	(0.91–1.90)	3	0.35	(0.09–1.36)
6─ < 12	148	101	1.15	(0.81–1.63)	11	0.88	(0.34–2.27)
12─ < 34	70	44	0.98	(0.61–1.57)	9	1.20	(0.42–3.44)
Stage							
Local	52	9	1.00	Ref.	9	1.00	Ref.
Advanced	330	256	9.05	(4.60–17.82)	19	0.80	(0.32–2.01)
Unknown	39	11	1.60	(0.66–3.89)	6	1.01	(0.32–3.23)
Pre-diagnostic statin use							
No	235	162	1.00	Ref.	20	1.00	Ref.
Yes	186	114	0.72	(0.56–0.93)	14	0.66	(0.30–1.43)
Pre-diagnostic ADM group							
Metformin	77	46	1.15	(0.74–1.79)	5	1.85	(0.44–7.73)
Other^a^	58	44	1.00	Ref.	4	1.00	Ref.
Metformin and other^a^	100	67	1.21	(0.82–1.80)	6	1.19	(0.32–4.38)
Insulin	82	59	1.49	(0.96–2.30)	7	1.61	(0.42–6.18)
None	104	60	0.69	(0.46–1.03)	12	1.48	(0.46–4.78)

All HRs were adjusted for the other factors in this table

^a^other oral antidiabetic medication

*OC* Ovarian cancer, *HR* Hazard ratio, *95% CI* 95% confidence interval

## Discussion

We found no statistically discernible differences in mortality from ovarian cancer or from other causes between the groups of ovarian cancer patients with T2D on different types of ADM in the three years before cancer diagnosis. However, pre-diagnostic use of statin was observed to be associated with an improved prognosis of OC, but this result must be interpreted with due caution. To our knowledge, this study is the first one to explore the association between statin use and ovarian cancer survival in women with T2D. Our study also has one of the largest study populations in addressing the relationship between ADM and ovarian cancer survival.

In studies carried out in vitro, statins have shown a favourable effect on cancer prognosis when combined with chemotherapy. In human ovarian cancer-cell lines, synergistic cytotoxicity is seen when combining fluvastatin and cisplatin. This has been suggested to be brought about by dysregulation of Ras-pathway proteins [33]. Simvastatin is especially cytotoxic when combined with carboplatin or paclitaxel at higher than physiologically used concentrations [34].

There are some previous cohort studies on statins and OC survival. Only two of them did not report a difference between statin users and non-users [20, 35]. In studies by Lavie et al. [16], Elmore et al. [17] and Vogel et al. [18], those ovarian cancer patients who used statins were observed to have better overall survival similarly to our study. In one of these studies, the suggested favourable effect on OC survival was seen only with lipophilic statins [18].

In a large population-based Danish study, post-diagnostic statin use was not found to be related to decreased all-cause or cancer-specific mortality among ovarian cancer patients unlike in our study [36]. However, a reduction in mortality from endometrioid and clear-cell ovarian cancer subtypes was observed in that study, although the limited numbers of these rare histological types of OC decreased the reliability of the results [36]. Mortality was also lower among those statin users who did not use low-dose aspirin or had started statin use after OC diagnosis [36]. In our study registered information on aspirin use was not available, as in Finland, aspirin is an over-the-counter drug.

The results of some previous studies have suggested that metformin use is associated with better survival in cases of ovarian cancer [6–8, 37] unlike in our study, whereas similarly to our findings, in one study such an association was not found [38]. The most recent study on metformin and OC survival also suggested that continuous use of metformin in women with T2D decreases the occurrence of relapses of ovarian cancer and ovarian cancer-related deaths [37]. In line with our findings, Garcia et al. found no association between metformin use and better overall survival [38]. However, the study populations in these two investigations were not limited to women with type 2 diabetes [37, 38].

Selection of the reference medication affects interpretation of the results. In our study, the reference group for metformin users was the group of users of other forms of oral ADM, which is relevant when addressing the possible influence of metformin on cancer survival in T2D patients. Using “no antidiabetic medication” as the reference group could lead to bias, as persons with T2D without any proper medication would represent a selective group with prognostic differences. In some previous studies the reference groups for metformin users have been non-users of metformin [8, 37, 38]. In a study carried out by Kumar et al. [6], the reference group comprised non-diabetic non-metformin users and women with diabetes who used insulin or other types of ADM.

A major strength of our study is the availability of reliable and comprehensive Finnish national registers. A unique personal identification code (PIC) is used in all registers involved. FinDM database covers whole Finland and therefore it is an exceptional resource. Data quality is usually considered to be high in Finnish registers, such as, for example, the Finnish Hospital Discharge Register [39]. The duration of diabetes is considered to be relatively accurately recorded in the FinDM database, even though there can be some minor errors connected to diet-controlled diabetes. In Finland, all forms of ADM and statins are prescribed by doctors and reimbursed by the Social Insurance Institution, and therefore data on the duration of use of medication is accurate. Also, the Finnish Cancer Registry is recognized to have high quality with regard to completeness and accuracy, 93% of cancer cases being microscopically verified [25].

In addition to the above, the size of our study cohort is greater than in previous studies addressing the roles of metformin and statins in connection with OC survival. In particular, the number of metformin users among OC patients was relatively large in our study compared with those in prior studies [6–8]. However, our study population is limited to women with T2D, and therefore the results can strictly be generalized only to women with T2D.

Obesity has been associated with poorer ovarian cancer prognosis [40], and, therefore, may be an important confounder in our study. However, our study lacked data on BMI. Also, the FinDM database does not contain information on aspects of life style, such as smoking, alcohol consumption, exercise or diet, which can also have an influence on ovarian cancer survival. Neither does FinDM database include measures regarding the severity of T2D, including data on HbA1c but duration of T2D and history of insulin use can be observed as surrogate indicators of the severity of T2D. In addition, we do not have data on cholesterol levels of the patients, and therefore we cannot be sure whether the observed association of mortality from OC with the use of statins is partly or wholly attributable to cholesterol levels, insofar as the latter were an independent prognostic marker of OC.

Comorbidities are not recorded in adequate completeness and detail in FinDM, and therefore not included in our study. It is known that statin use is linked to heart diseases [13] and is thus related to mortality from causes other than cancer. Despite our relatively large cohort, the number of deaths from causes other than cancer was small with the consequence that our estimation results on this component of mortality are highly unreliable with a wide margin of error.

The Finnish Cancer Registry also contains some information about the treatment of cancer, but the data are not comprehensive or complete enough and thus were not included in our study. However, the national ovarian cancer treatment schedule has guidelines concerning surgery and first-line chemotherapy, and these guidelines did not change during the study period [41].

In pharmacoepidemiological studies exposure assessment can never be completely free from misclassification. Therefore, some information bias as to the use of drugs under study is to be expected. However, the concordance between self-reported medication use and information contained in the prescription register has been shown to be good [42]. The reimbursement connected with the costs of ADM and statins also strengthens the reliability of our data. However, drugs dispensed in hospitals and outpatient clinics are not covered by the Register on Reimbursed Drug purchases and therefore we lack data on medication used by the small proportion of patients who were treated in healthcare facilities.

Socioeconomic differences might be associated with statin use. It has been found that in patients with lower income, the use of statins is 10% lower compared with the overall level [13]. In our study, data on socioeconomic status was not available and therefore not adjusted for in our results, and this could lead to a healthy-user bias in statin users.

## Conclusion

Our findings are inconclusive as regards an association between metformin and OC survival. However, there is some evidence of improved prognosis of ovarian cancer with pre-diagnostic statin use. Ovarian cancer is a rare disease associated with high mortality, and more research is needed to find new forms of medication to improve its prognosis.

## Additional files

Additional file 1: ICD-10 and ICD-O-3 codes for epithelial ovarian cancer and different histological types. (XLSX 10 kb)

Additional file 2: ATC codes for different types of oral ADM and statins. (XLSX 10 kb)

Additional file 3: Label: Estimated HRs (with 95% CIs) of OC death in relation to cumulative use of medications. Fitted curves are cubic splines, with inner knots at 1.5 and 3 years, estimated from a mutually adjusted Cox regression model. *OC* ovarian cancer, *HR* hazard ratio, *95% CI* 95% confidence interval*, DDD* defined daily dose. (PDF 251 kb)

## Abbreviations

ADM: Antidiabetic medication
ATC: Anatomical therapeutic chemical
BMI: Body mass index
CI: Confidence interval
DDD: Defined daily doses
FinDM: Nationwide diabetes database
HMG-CoA: 3-hydroxy-3-methylglutaryl-CoA
HR: Hazard ratio
ICD-10: International statistical classification of diseases and related health problems, 10th revision
ICD-O-3: International classification of diseases for oncology, third edition
OC: Ovarian cancer
PIC: Personal identification code
T2D: Type 2 diabetes
WHO: World Health Organization
