# Supplementary figures and images for: Prognosis of ovarian cancer in women with type 2 diabetes using metformin and other forms of antidiabetic medication or statins: a retrospective cohort study
Source: BMC Cancer. 2018 Jul 28;18:767. doi: 10.1186/s12885-018-4676-z (PMC6064082; doi:10.1186/s12885-018-4676-z)

A. Insulin

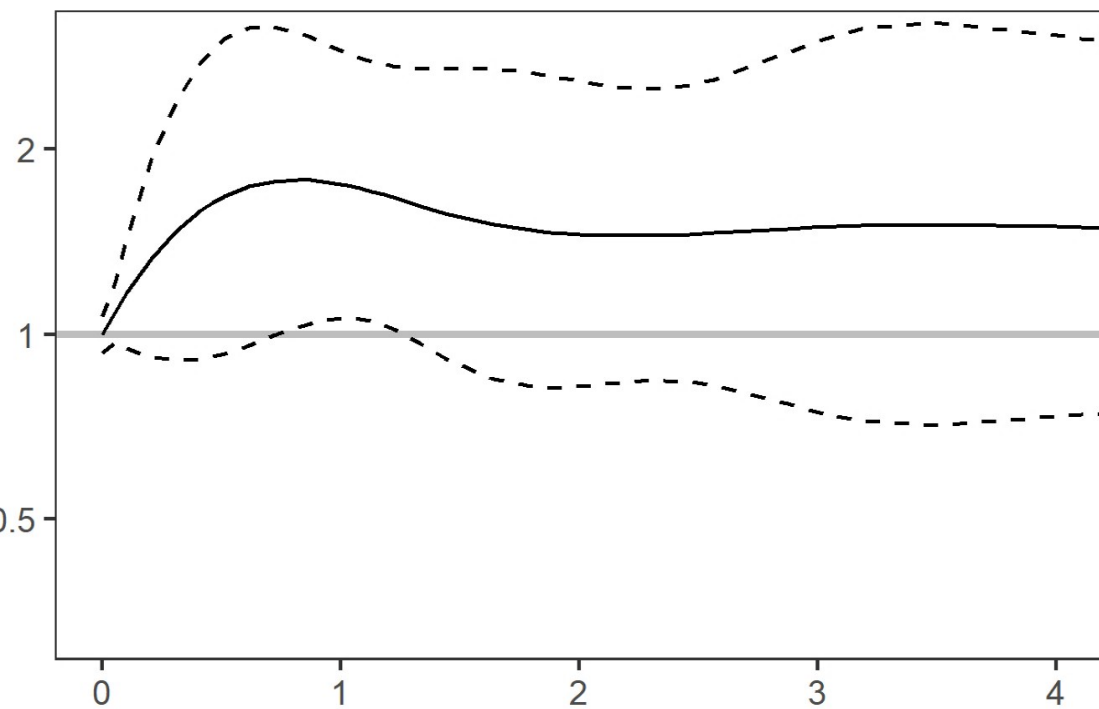

B. Metformin

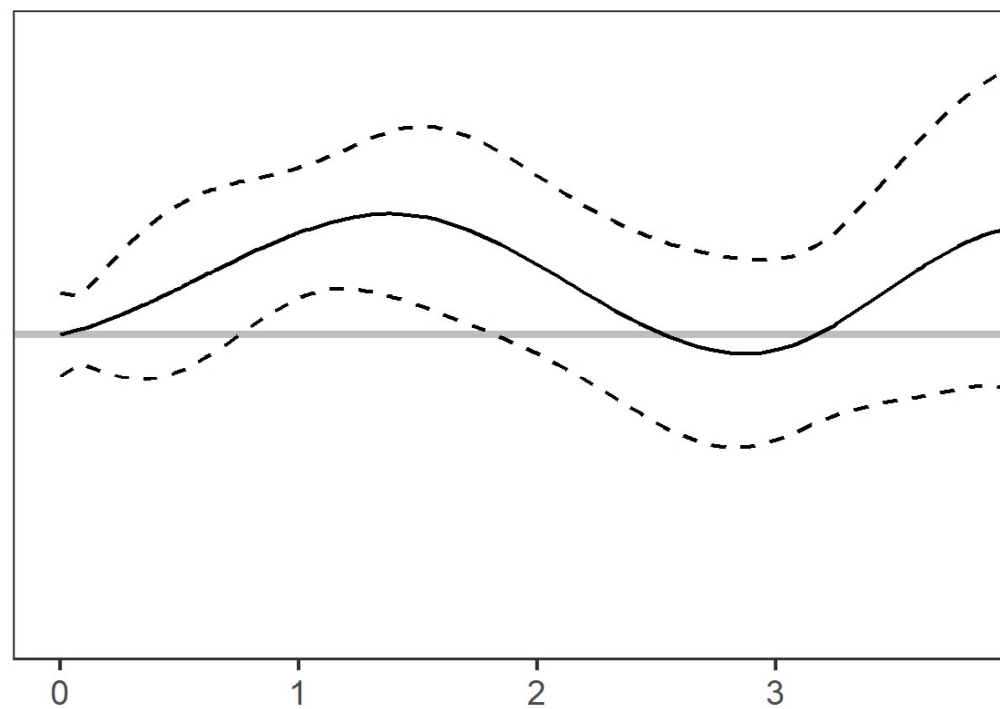

C. Other oral

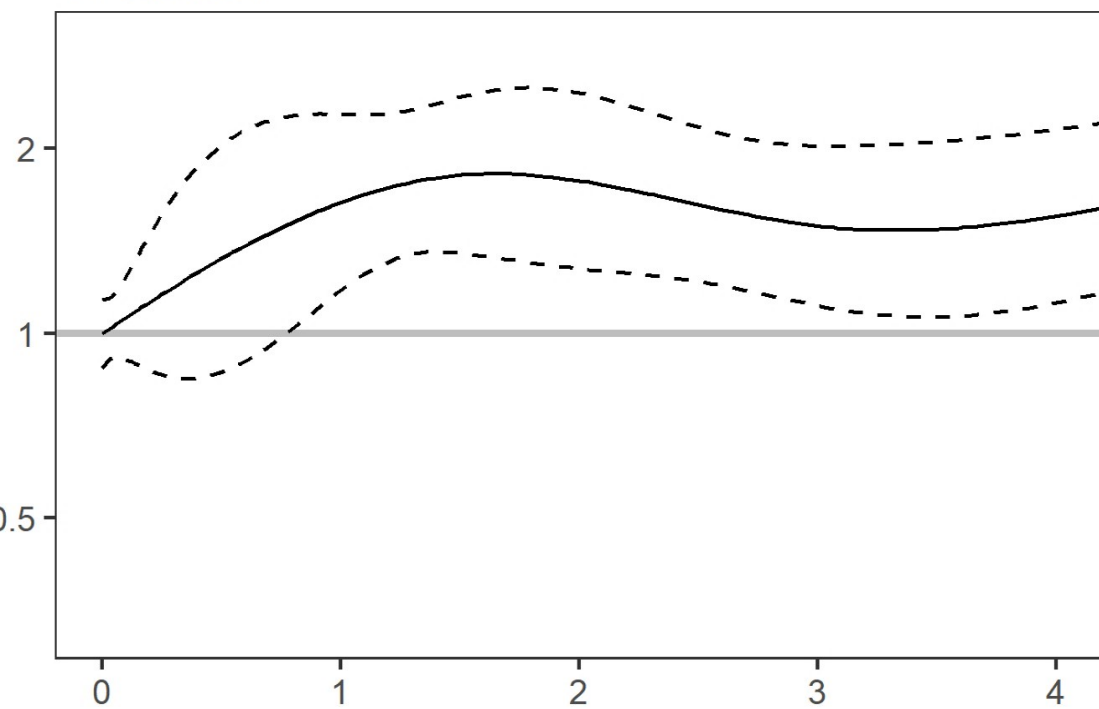

D. Statin

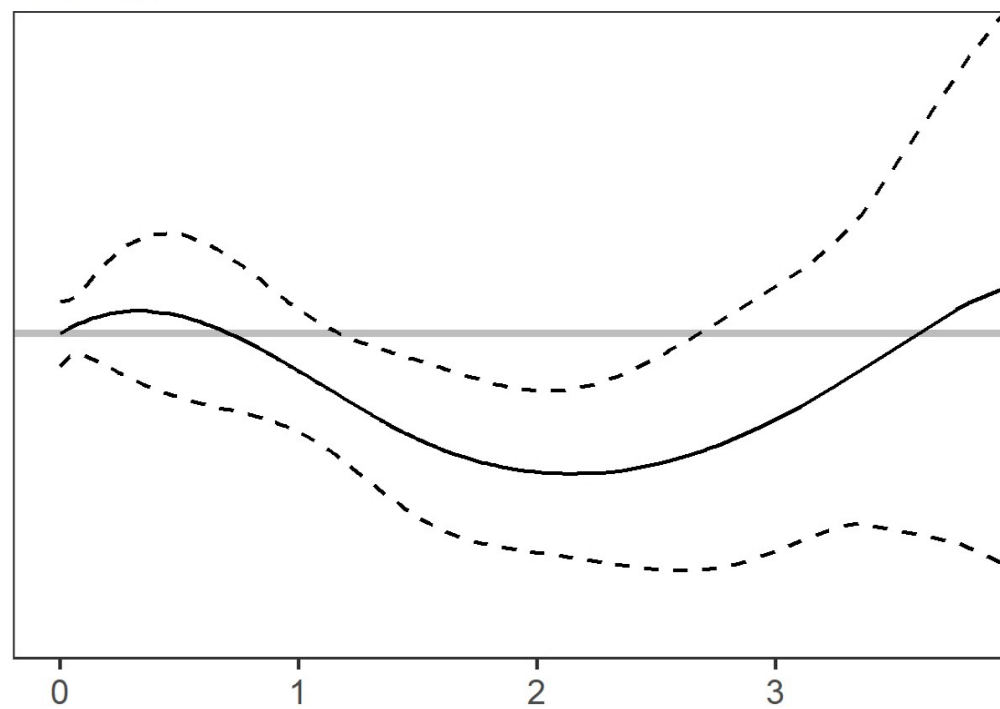

Years of DDDs

Supplement: Supplementary file 3 — Label: Estimated HRs (with 95% CIs) of OC death in relation to cumulative use of medications. Fitted curves are cubic splines, with inner knots at 1.5 and 3 years, estimated from a mutually adjusted Cox regression model. OC ovarian cancer, HR hazard ratio, 95% CI 95% confidence interval, DDD defined daily dose. (PDF 251 kb) [file 12885_2018_4676_MOESM3_ESM.pdf]
